# Methyl (2*Z*)-2-{[*N*-(2-formyl­phen­yl)-4-methyl­benzene­sulfonamido]­meth­yl}-3-(naphthalen-1-yl)prop-2-enoate

**DOI:** 10.1107/S160053681200058X

**Published:** 2012-01-11

**Authors:** R. Madhanraj, S. Murugavel, D. Kannan, M. Bakthadoss

**Affiliations:** a Department of Physics, Ranipettai Engineering College, Thenkadapathangal, Walaja 632 513, India; b Department of Physics, Thanthai Periyar Government Institute of Technology, Vellore 632 002, India; c Department of Organic Chemistry, University of Madras, Maraimalai Campus, Chennai 600 025, India; d Department of Organic Chemistry, University of Madras, Maraimalai Campus, Chennai 600 025, India.

## Abstract

In the title compound, C_29_H_25_NO_5_S, the sulfonyl-bound benzene ring forms dihedral angles of 42.1 (1) and 48.5 (1)°, respectively, with the formyl-substituted benzene ring and the naphthalene residue. In the crystal, pairs of C—H⋯O inter­actions lead to the formation of *R*_2_^2^(10) inversion dimers, which are linked by further C—H⋯O inter­actions into supra­molecular tapes running along [100]. The crystal packing is further stabilized by C—H⋯π inter­actions.

## Related literature

For background to the pharmacological uses of sulfonamides, see: Korolkovas (1988 ▶); Mandell & Sande (1992 ▶). For resonance effects of acrylate, see: Merlino (1971 ▶); Varghese *et al.* (1986 ▶). For related structures, see: Madhanraj *et al.* (2011 ▶); Aziz-ur-Rehman *et al.* (2010 ▶).
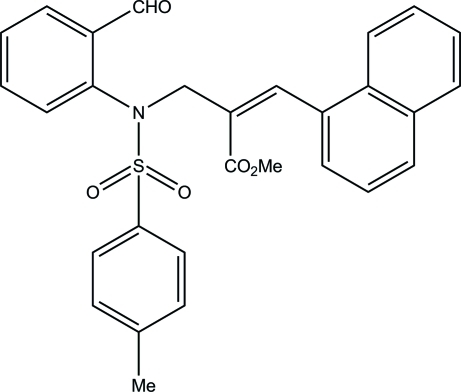


## Experimental

### 

#### Crystal data

C_29_H_25_NO_5_S
*M* *_r_* = 499.56
Triclinic, 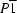

*a* = 8.0162 (3) Å
*b* = 12.0887 (5) Å
*c* = 13.8703 (6) Å
α = 107.788 (2)°
β = 90.068 (1)°
γ = 93.446 (2)°
*V* = 1277.27 (9) Å^3^
*Z* = 2
Mo *K*α radiation
μ = 0.17 mm^−1^
*T* = 293 K
0.23 × 0.21 × 0.16 mm

#### Data collection

Bruker APEXII CCD diffractometer
Absorption correction: multi-scan (*SADABS*; Sheldrick, 1996 ▶) *T*_min_ = 0.963, *T*_max_ = 0.974
22485 measured reflections
4932 independent reflections
3520 reflections with *I* > 2σ(*I*)
*R*_int_ = 0.031

#### Refinement

*R*[*F* ^2^ > 2σ(*F* ^2^)] = 0.040
*wR*(*F* ^2^) = 0.116
*S* = 1.01
4932 reflections
327 parameters
H-atom parameters constrained
Δρ_max_ = 0.19 e Å^−3^
Δρ_min_ = −0.23 e Å^−3^

### 

Data collection: *APEX2* (Bruker, 2004 ▶); cell refinement: *APEX2* and *SAINT* (Bruker, 2004 ▶); data reduction: *SAINT* and *XPREP* (Bruker, 2004 ▶); program(s) used to solve structure: *SHELXS97* (Sheldrick, 2008 ▶); program(s) used to refine structure: *SHELXL97* (Sheldrick, 2008 ▶); molecular graphics: *ORTEP-3* (Farrugia (1997 ▶); software used to prepare material for publication: *SHELXL97* and *PLATON* (Spek, 2009 ▶).

## Supplementary Material

Crystal structure: contains datablock(s) global, I. DOI: 10.1107/S160053681200058X/bt5780sup1.cif

Structure factors: contains datablock(s) I. DOI: 10.1107/S160053681200058X/bt5780Isup2.hkl

Supplementary material file. DOI: 10.1107/S160053681200058X/bt5780Isup3.cml

Additional supplementary materials:  crystallographic information; 3D view; checkCIF report

## Figures and Tables

**Table 1:** Hydrogen-bond geometry (Å, °) *Cg* is the centroid of the C22/C23/C26–C29 ring.

*D*—H⋯*A*	*D*—H	H⋯*A*	*D*⋯*A*	*D*—H⋯*A*
C25—H25*A*⋯O4^i^	0.96	2.50	3.462 (3)	177
C10—H10⋯O2^ii^	0.93	2.44	3.305 (2)	155
C17—H17⋯*Cg*^iii^	0.93	2.78	3.528 (2)	138

Symmetry codes: (i) 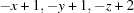; (ii) 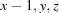; (iii) 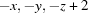.

**Fig. 1.**
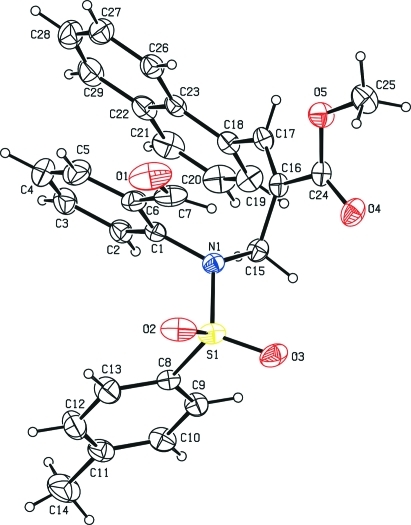
The molecular structure of the title compound with the atom numbering scheme. Displacement ellipsoids are drawn at the 20% probability level. H atoms are presented as a small spheres of arbitrary radius.

**Fig. 2.**
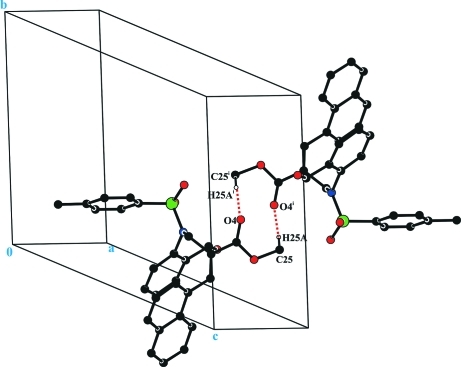
Part of the crystal structure of the title compound showing C—H···O intermolecular hydrogen bonds (dotted lines) generating R22(10) centrosymmetric dimer. [Symmetry code: (i) 1-x, 1-y, 2-z].

**Fig. 3.**
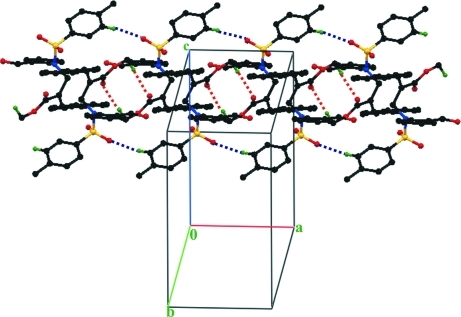
Supramolecular tape formation in (I) whereby centrosymmetrc R22(10) dimeric aggregates sustained by C—H···O (red dashed lines) contacts are linked via C—H···O contacts (blue dashed lines) along [1 0 0].

**Fig. 4.**
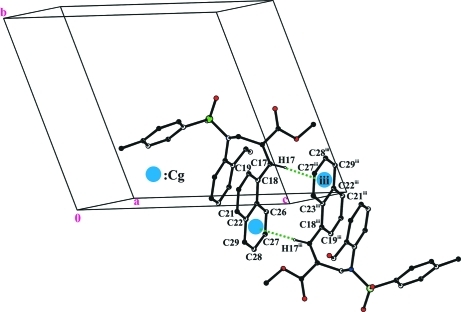
Part of the crystal structure of the title compound showing the formation of centrosymmetric dimer by means of symmetry–related C-H···π(arene) hydrogen bonds. [Symmetry code: (iii) -x, -y, 2-z].

## supplementary crystallographic information

### Comment

Sulfonamide drugs are widely used for the treatment of certain infections caused by Gram-positive and Gram-negative microorganisms, some fungi, and certain protozoa (Korolkovas, 1988, Mandell & Sande, 1992). In view of this biological importance, the crystal structure of the title compound has been determined and the results are presented here.

Fig. 1. shows a displacement ellipsoid plot of the title compound, with the atom numbering scheme. The significant difference in length of the C24—O5 = 1.332 (2) Å and C25—O5 = 1.443 (2) Å bonds is attributed to a partial contribution from the O^-^–C = O^+^–C resonance structure of the O4═C24—O5—C25 group (Merlino, 1971). This feature, commonly observed in the carboxylic ester group of the substituents in various compounds gives average values of 1.340 Å and 1.447 Å respectively for these bonds (Varghese *et al.,* 1986). The sum of bond angles around N1 (350.6°) indicates that N1 is in *sp*^2^ hybridization. The sulfonyl–bound benzene (C8–C13) ring forms dihedral angles of 42.1 (1)° and 48.5 (1)° respectively, with the formyl phenyl (C1–C6) and naphthalene (C18–C23/C26–C29) rings. The dihedral angle between formyl phenyl and naphthalene rings is 8.9 (1)°. The geometric parameters of the title molecule agree well with those reported for similar structures (Madhanraj *et al.*, 2011; Aziz-ur-Rehman *et al.*, 2010).

The crystal packing is stabilized by intermolecular C—H···O hydrogen bonds. The molecules at *x, y, z* and *1-x, 1-y, 2-z* are linked by C25—H25A···O4 hydrogen bonds into cyclic centrosymmetric *R*_2_^2^(10) dimers (Fig. 2). These dimers are linked by C10—H10···O2 hydrogen bonds forming supramolecular tapes running along the [100] directions (Fig. 3). The crystal packing is further stabilized by C-H···π interactions between H17 atom and the ring (C22/C23/C26–C29) at *-x, -y, 2-z* combine two molecules into a centrosymmetric inverted dimer (Table. 1 and Fig. 4).

### Experimental

A solution of *N*-(2-formylphenyl)-4-methylbenzene-1-sulfonamide (1 mmol, 0.275 g) and potassium carbonate (1.5 mmol, 0.207 g) in acetonitrile solvent was stirred for 15 minutes at room temperature. To this solution, methyl(2*E*-2-(bromomethyl)-3-(naphthalen-1-yl) prop-2-enoate (1.2 mmol, 0.366 g) was added dropwise till the addition is complete. After the completion of the reaction, as indicated by TLC, acetonitile was evaporated. ETOAc (15 ml) were added to the crude mass. The organic layer was dried over anhydrous sodium sulfate. Removal of solvent led to the crude product, which was purified through pad of silica gel (100-200 mesh) using ethylacetate and hexanes (1:9) as solvents. The pure title compound was obtained as a colourless solid (0.450 g, 90% yield). Recrystallization was carried out using ethylacetate as solvent.

### Refinement

All the H atoms were positioned geometrically, with C–H = 0.93–0.97 Å and constrained to ride on their parent atom, with *U*_iso_(H) =1.5*U*_eq_ for methyl H atoms and 1.2*U*_eq_(C) for other H atoms.

### Crystal data

**Table:**

C_29_H_25_NO_5_S	*Z* = 2
*M**_r_* = 499.56	*F*(000) = 524
Triclinic, *P*1	*D*_x_ = 1.299 Mg m^−^^3^
Hall symbol: -P 1	Mo *K*α radiation, λ = 0.71073 Å
*a* = 8.0162 (3) Å	Cell parameters from 4958 reflections
*b* = 12.0887 (5) Å	θ = 1.5–25.9°
*c* = 13.8703 (6) Å	µ = 0.17 mm^−^^1^
α = 107.788 (2)°	*T* = 293 K
β = 90.068 (1)°	Block, colourless
γ = 93.446 (2)°	0.23 × 0.21 × 0.16 mm
*V* = 1277.27 (9) Å^3^	

### Data collection

**Table:**

Bruker APEXII CCD diffractometer	4932 independent reflections
Radiation source: fine-focus sealed tube	3520 reflections with *I* > 2σ(*I*)
graphite	*R*_int_ = 0.031
Detector resolution: 10.0 pixels mm^-1^	θ_max_ = 25.9°, θ_min_ = 2.6°
ω scans	*h* = −9→9
Absorption correction: multi-scan (*SADABS*; Sheldrick, 1996)	*k* = −14→14
*T*_min_ = 0.963, *T*_max_ = 0.974	*l* = −17→16
22485 measured reflections	

### Refinement

**Table:**

Refinement on *F*^2^	Primary atom site location: structure-invariant direct methods
Least-squares matrix: full	Secondary atom site location: difference Fourier map
*R*[*F*^2^ > 2σ(*F*^2^)] = 0.040	Hydrogen site location: inferred from neighbouring sites
*wR*(*F*^2^) = 0.116	H-atom parameters constrained
*S* = 1.01	*w* = 1/[σ^2^(*F*_o_^2^) + (0.0565*P*)^2^ + 0.1916*P*] where *P* = (*F*_o_^2^ + 2*F*_c_^2^)/3
4932 reflections	(Δ/σ)_max_ = 0.001
327 parameters	Δρ_max_ = 0.19 e Å^−^^3^
0 restraints	Δρ_min_ = −0.23 e Å^−^^3^

### Special details

**Table:**

Geometry. All esds (except the esd in the dihedral angle between two l.s. planes) are estimated using the full covariance matrix. The cell esds are taken into account individually in the estimation of esds in distances, angles and torsion angles; correlations between esds in cell parameters are only used when they are defined by crystal symmetry. An approximate (isotropic) treatment of cell esds is used for estimating esds involving l.s. planes.
Refinement. Refinement of F^2^ against ALL reflections. The weighted R-factor wR and goodness of fit S are based on F^2^, conventional R-factors R are based on F, with F set to zero for negative F^2^. The threshold expression of F^2^ > 2sigma(F^2^) is used only for calculating R-factors(gt) etc. and is not relevant to the choice of reflections for refinement. R-factors based on F^2^ are statistically about twice as large as those based on F, and R- factors based on ALL data will be even larger.

### Fractional atomic coordinates and isotropic or equivalent isotropic displacement parameters (Å^2^)

**Table:**

	*x*	*y*	*z*	*U*_iso_*/*U*_eq_	
S1	0.16584 (5)	0.44112 (4)	0.73002 (4)	0.05990 (17)	
N1	0.15470 (16)	0.34007 (12)	0.78747 (11)	0.0497 (3)	
O5	0.34851 (16)	0.31525 (12)	1.04708 (11)	0.0702 (4)	
C8	−0.0019 (2)	0.41133 (15)	0.64232 (14)	0.0507 (4)	
C1	0.1799 (2)	0.22349 (15)	0.72522 (12)	0.0487 (4)	
O2	0.31605 (15)	0.42505 (14)	0.67383 (13)	0.0834 (5)	
O3	0.14348 (18)	0.54886 (11)	0.80521 (12)	0.0800 (4)	
O4	0.27092 (17)	0.47877 (12)	1.02354 (12)	0.0765 (4)	
C23	−0.0828 (2)	0.01039 (16)	0.87084 (13)	0.0548 (4)	
C18	−0.0968 (2)	0.13048 (16)	0.92080 (13)	0.0553 (4)	
C16	0.1067 (2)	0.30378 (15)	0.94720 (12)	0.0507 (4)	
C2	0.0452 (2)	0.14657 (17)	0.68456 (14)	0.0610 (5)	
H2	−0.0632	0.1698	0.6979	0.073*	
C17	0.0536 (2)	0.20561 (16)	0.96421 (13)	0.0553 (4)	
H17	0.1187	0.1813	1.0086	0.066*	
C9	−0.1560 (2)	0.44809 (17)	0.67467 (15)	0.0602 (5)	
H9	−0.1710	0.4899	0.7422	0.072*	
C15	0.0337 (2)	0.35257 (15)	0.87105 (14)	0.0551 (4)	
H15A	−0.0719	0.3108	0.8447	0.066*	
H15B	0.0138	0.4340	0.9023	0.066*	
C6	0.3421 (2)	0.18822 (17)	0.70641 (15)	0.0617 (5)	
C24	0.2488 (2)	0.37625 (16)	1.00878 (13)	0.0544 (4)	
C11	−0.2700 (3)	0.36137 (19)	0.50739 (17)	0.0692 (5)	
C26	0.0718 (3)	−0.04169 (18)	0.85611 (15)	0.0659 (5)	
H26	0.1698	0.0040	0.8795	0.079*	
C22	−0.2299 (3)	−0.06165 (18)	0.83382 (15)	0.0681 (5)	
C21	−0.3850 (3)	−0.0120 (2)	0.8509 (2)	0.0887 (7)	
H21	−0.4820	−0.0587	0.8274	0.106*	
O1	0.63132 (19)	0.24067 (17)	0.72916 (18)	0.1204 (7)	
C13	0.0203 (3)	0.3499 (2)	0.54323 (16)	0.0786 (6)	
H13	0.1250	0.3251	0.5209	0.094*	
C7	0.4908 (2)	0.2635 (2)	0.7536 (2)	0.0795 (6)	
H7	0.4748	0.3321	0.8050	0.095*	
C10	−0.2882 (2)	0.42301 (19)	0.60714 (16)	0.0687 (5)	
H10	−0.3926	0.4484	0.6296	0.082*	
C19	−0.2510 (3)	0.1742 (2)	0.93437 (17)	0.0738 (6)	
H19	−0.2600	0.2532	0.9665	0.089*	
C3	0.0714 (3)	0.0355 (2)	0.62431 (17)	0.0826 (7)	
H3	−0.0193	−0.0168	0.5979	0.099*	
C29	−0.2140 (4)	−0.1803 (2)	0.78207 (17)	0.0890 (8)	
H29	−0.3091	−0.2276	0.7551	0.107*	
C5	0.3645 (3)	0.0768 (2)	0.6432 (2)	0.0884 (7)	
H5	0.4722	0.0532	0.6281	0.106*	
C14	−0.4162 (3)	0.3322 (3)	0.4336 (2)	0.1124 (10)	
H14A	−0.4909	0.3942	0.4516	0.169*	
H14B	−0.4744	0.2613	0.4357	0.169*	
H14C	−0.3765	0.3227	0.3664	0.169*	
C27	0.0799 (3)	−0.1572 (2)	0.80839 (17)	0.0830 (7)	
H27	0.1828	−0.1903	0.8008	0.100*	
C25	0.4827 (2)	0.3807 (2)	1.11357 (17)	0.0789 (6)	
H25A	0.5534	0.4210	1.0777	0.118*	
H25B	0.5469	0.3286	1.1362	0.118*	
H25C	0.4369	0.4361	1.1710	0.118*	
C20	−0.3964 (3)	0.1018 (2)	0.9005 (2)	0.0898 (7)	
H20	−0.5008	0.1324	0.9124	0.108*	
C28	−0.0645 (4)	−0.2266 (2)	0.77079 (19)	0.0961 (8)	
H28	−0.0576	−0.3056	0.7376	0.115*	
C12	−0.1141 (3)	0.3253 (2)	0.47704 (17)	0.0897 (7)	
H12	−0.0991	0.2830	0.4097	0.108*	
C4	0.2318 (4)	0.0018 (2)	0.60314 (19)	0.0966 (8)	
H4	0.2491	−0.0728	0.5612	0.116*	

### Atomic displacement parameters (Å^2^)

**Table:**

	*U*^11^	*U*^22^	*U*^33^	*U*^12^	*U*^13^	*U*^23^
S1	0.0404 (2)	0.0595 (3)	0.0869 (4)	−0.00416 (19)	−0.0106 (2)	0.0344 (3)
N1	0.0424 (7)	0.0498 (8)	0.0576 (9)	0.0038 (6)	−0.0025 (6)	0.0174 (7)
O5	0.0600 (8)	0.0685 (8)	0.0787 (9)	−0.0140 (6)	−0.0221 (7)	0.0211 (7)
C8	0.0427 (9)	0.0513 (10)	0.0631 (11)	0.0025 (7)	0.0010 (8)	0.0252 (9)
C1	0.0480 (9)	0.0540 (10)	0.0472 (9)	0.0049 (8)	−0.0019 (7)	0.0196 (8)
O2	0.0380 (7)	0.1093 (11)	0.1291 (13)	0.0009 (7)	0.0055 (7)	0.0759 (10)
O3	0.0795 (9)	0.0463 (7)	0.1094 (12)	−0.0062 (6)	−0.0360 (8)	0.0186 (8)
O4	0.0716 (9)	0.0545 (8)	0.0906 (10)	−0.0055 (7)	−0.0083 (8)	0.0049 (7)
C23	0.0625 (11)	0.0603 (11)	0.0440 (9)	−0.0096 (9)	−0.0043 (8)	0.0222 (9)
C18	0.0530 (10)	0.0621 (11)	0.0513 (10)	−0.0062 (8)	−0.0016 (8)	0.0202 (9)
C16	0.0454 (9)	0.0539 (10)	0.0464 (9)	0.0010 (8)	0.0030 (7)	0.0061 (8)
C2	0.0609 (11)	0.0650 (12)	0.0562 (11)	−0.0023 (9)	−0.0071 (9)	0.0183 (10)
C17	0.0529 (10)	0.0618 (11)	0.0470 (10)	−0.0031 (8)	−0.0031 (8)	0.0119 (9)
C9	0.0449 (10)	0.0726 (12)	0.0624 (11)	0.0091 (9)	0.0000 (8)	0.0186 (10)
C15	0.0441 (9)	0.0538 (10)	0.0635 (11)	0.0063 (8)	0.0013 (8)	0.0116 (9)
C6	0.0578 (11)	0.0662 (12)	0.0699 (12)	0.0160 (9)	0.0065 (9)	0.0315 (10)
C24	0.0489 (10)	0.0552 (11)	0.0511 (10)	−0.0014 (8)	0.0051 (8)	0.0053 (9)
C11	0.0646 (13)	0.0755 (13)	0.0728 (14)	−0.0040 (10)	−0.0153 (10)	0.0321 (11)
C26	0.0715 (13)	0.0700 (13)	0.0585 (11)	−0.0008 (10)	0.0024 (10)	0.0237 (10)
C22	0.0767 (14)	0.0709 (13)	0.0592 (12)	−0.0224 (11)	−0.0120 (10)	0.0288 (10)
C21	0.0653 (15)	0.1012 (19)	0.1063 (19)	−0.0308 (13)	−0.0220 (13)	0.0485 (16)
O1	0.0474 (9)	0.1394 (16)	0.206 (2)	0.0216 (9)	0.0138 (11)	0.0966 (15)
C13	0.0608 (13)	0.1092 (18)	0.0691 (14)	0.0235 (12)	0.0120 (11)	0.0287 (13)
C7	0.0494 (12)	0.0868 (15)	0.1220 (19)	0.0099 (10)	−0.0032 (12)	0.0603 (14)
C10	0.0453 (10)	0.0889 (15)	0.0774 (14)	0.0068 (9)	−0.0011 (10)	0.0330 (12)
C19	0.0590 (12)	0.0762 (14)	0.0869 (15)	0.0012 (10)	0.0072 (11)	0.0265 (12)
C3	0.1072 (19)	0.0684 (14)	0.0654 (13)	−0.0105 (13)	−0.0183 (13)	0.0134 (11)
C29	0.120 (2)	0.0722 (16)	0.0704 (15)	−0.0366 (15)	−0.0162 (14)	0.0236 (13)
C5	0.0900 (18)	0.0843 (17)	0.0969 (18)	0.0357 (14)	0.0201 (14)	0.0312 (14)
C14	0.106 (2)	0.128 (2)	0.107 (2)	−0.0087 (17)	−0.0507 (17)	0.0438 (18)
C27	0.1054 (19)	0.0709 (15)	0.0753 (15)	0.0135 (13)	0.0159 (13)	0.0248 (12)
C25	0.0586 (12)	0.0940 (16)	0.0771 (14)	−0.0224 (11)	−0.0221 (10)	0.0208 (12)
C20	0.0501 (12)	0.1022 (19)	0.126 (2)	−0.0058 (12)	−0.0021 (13)	0.0500 (17)
C28	0.145 (3)	0.0615 (14)	0.0779 (16)	−0.0086 (17)	0.0134 (17)	0.0187 (12)
C12	0.0936 (18)	0.115 (2)	0.0557 (13)	0.0193 (14)	−0.0014 (12)	0.0162 (13)
C4	0.133 (2)	0.0681 (15)	0.0801 (17)	0.0262 (16)	0.0096 (16)	0.0062 (13)

### Geometric parameters (Å, °)

**Table:**

S1—O3	1.4194 (15)	C11—C14	1.508 (3)
S1—O2	1.4258 (14)	C26—C27	1.356 (3)
S1—N1	1.6483 (14)	C26—H26	0.9300
S1—C8	1.7572 (18)	C22—C21	1.402 (3)
N1—C1	1.436 (2)	C22—C29	1.408 (3)
N1—C15	1.490 (2)	C21—C20	1.345 (3)
O5—C24	1.332 (2)	C21—H21	0.9300
O5—C25	1.443 (2)	O1—C7	1.200 (2)
C8—C13	1.366 (3)	C13—C12	1.373 (3)
C8—C9	1.367 (2)	C13—H13	0.9300
C1—C2	1.381 (2)	C7—H7	0.9300
C1—C6	1.393 (2)	C10—H10	0.9300
O4—C24	1.195 (2)	C19—C20	1.404 (3)
C23—C26	1.411 (3)	C19—H19	0.9300
C23—C18	1.416 (2)	C3—C4	1.377 (3)
C23—C22	1.419 (3)	C3—H3	0.9300
C18—C19	1.364 (3)	C29—C28	1.342 (4)
C18—C17	1.474 (2)	C29—H29	0.9300
C16—C17	1.325 (2)	C5—C4	1.356 (4)
C16—C24	1.488 (2)	C5—H5	0.9300
C16—C15	1.492 (2)	C14—H14A	0.9600
C2—C3	1.376 (3)	C14—H14B	0.9600
C2—H2	0.9300	C14—H14C	0.9600
C17—H17	0.9300	C27—C28	1.389 (3)
C9—C10	1.371 (3)	C27—H27	0.9300
C9—H9	0.9300	C25—H25A	0.9600
C15—H15A	0.9700	C25—H25B	0.9600
C15—H15B	0.9700	C25—H25C	0.9600
C6—C5	1.387 (3)	C20—H20	0.9300
C6—C7	1.477 (3)	C28—H28	0.9300
C11—C10	1.369 (3)	C12—H12	0.9300
C11—C12	1.372 (3)	C4—H4	0.9300
			
O3—S1—O2	119.86 (10)	C21—C22—C23	118.7 (2)
O3—S1—N1	106.84 (9)	C29—C22—C23	118.6 (2)
O2—S1—N1	106.02 (8)	C20—C21—C22	121.4 (2)
O3—S1—C8	108.59 (8)	C20—C21—H21	119.3
O2—S1—C8	107.37 (9)	C22—C21—H21	119.3
N1—S1—C8	107.59 (8)	C8—C13—C12	119.1 (2)
C1—N1—C15	116.53 (13)	C8—C13—H13	120.5
C1—N1—S1	116.14 (11)	C12—C13—H13	120.5
C15—N1—S1	117.98 (11)	O1—C7—C6	123.5 (2)
C24—O5—C25	116.36 (15)	O1—C7—H7	118.3
C13—C8—C9	120.22 (18)	C6—C7—H7	118.3
C13—C8—S1	120.42 (14)	C11—C10—C9	121.44 (19)
C9—C8—S1	119.35 (15)	C11—C10—H10	119.3
C2—C1—C6	120.04 (17)	C9—C10—H10	119.3
C2—C1—N1	120.62 (15)	C18—C19—C20	121.0 (2)
C6—C1—N1	119.33 (15)	C18—C19—H19	119.5
C26—C23—C18	122.86 (17)	C20—C19—H19	119.5
C26—C23—C22	118.01 (19)	C2—C3—C4	120.0 (2)
C18—C23—C22	119.13 (18)	C2—C3—H3	120.0
C19—C18—C23	119.57 (17)	C4—C3—H3	120.0
C19—C18—C17	120.37 (18)	C28—C29—C22	121.4 (2)
C23—C18—C17	119.91 (16)	C28—C29—H29	119.3
C17—C16—C24	119.54 (17)	C22—C29—H29	119.3
C17—C16—C15	125.68 (16)	C4—C5—C6	121.0 (2)
C24—C16—C15	114.77 (16)	C4—C5—H5	119.5
C3—C2—C1	119.9 (2)	C6—C5—H5	119.5
C3—C2—H2	120.0	C11—C14—H14A	109.5
C1—C2—H2	120.0	C11—C14—H14B	109.5
C16—C17—C18	127.39 (17)	H14A—C14—H14B	109.5
C16—C17—H17	116.3	C11—C14—H14C	109.5
C18—C17—H17	116.3	H14A—C14—H14C	109.5
C8—C9—C10	119.69 (19)	H14B—C14—H14C	109.5
C8—C9—H9	120.2	C26—C27—C28	120.4 (2)
C10—C9—H9	120.2	C26—C27—H27	119.8
N1—C15—C16	108.06 (13)	C28—C27—H27	119.8
N1—C15—H15A	110.1	O5—C25—H25A	109.5
C16—C15—H15A	110.1	O5—C25—H25B	109.5
N1—C15—H15B	110.1	H25A—C25—H25B	109.5
C16—C15—H15B	110.1	O5—C25—H25C	109.5
H15A—C15—H15B	108.4	H25A—C25—H25C	109.5
C5—C6—C1	118.6 (2)	H25B—C25—H25C	109.5
C5—C6—C7	118.7 (2)	C21—C20—C19	120.1 (2)
C1—C6—C7	122.60 (18)	C21—C20—H20	120.0
O4—C24—O5	123.14 (17)	C19—C20—H20	120.0
O4—C24—C16	123.83 (18)	C29—C28—C27	120.4 (2)
O5—C24—C16	113.03 (16)	C29—C28—H28	119.8
C10—C11—C12	117.65 (19)	C27—C28—H28	119.8
C10—C11—C14	121.4 (2)	C11—C12—C13	121.9 (2)
C12—C11—C14	121.0 (2)	C11—C12—H12	119.0
C27—C26—C23	121.1 (2)	C13—C12—H12	119.0
C27—C26—H26	119.4	C5—C4—C3	120.3 (2)
C23—C26—H26	119.4	C5—C4—H4	119.9
C21—C22—C29	122.7 (2)	C3—C4—H4	119.9
			
O3—S1—N1—C1	−177.99 (11)	C25—O5—C24—C16	−175.82 (15)
O2—S1—N1—C1	−49.07 (14)	C17—C16—C24—O4	−154.66 (19)
C8—S1—N1—C1	65.57 (13)	C15—C16—C24—O4	24.1 (2)
O3—S1—N1—C15	36.52 (13)	C17—C16—C24—O5	24.6 (2)
O2—S1—N1—C15	165.44 (12)	C15—C16—C24—O5	−156.62 (15)
C8—S1—N1—C15	−79.92 (13)	C18—C23—C26—C27	179.60 (18)
O3—S1—C8—C13	149.02 (17)	C22—C23—C26—C27	−0.3 (3)
O2—S1—C8—C13	18.05 (19)	C26—C23—C22—C21	177.87 (18)
N1—S1—C8—C13	−95.69 (17)	C18—C23—C22—C21	−2.0 (3)
O3—S1—C8—C9	−32.37 (17)	C26—C23—C22—C29	−1.7 (3)
O2—S1—C8—C9	−163.34 (14)	C18—C23—C22—C29	178.44 (17)
N1—S1—C8—C9	82.92 (15)	C29—C22—C21—C20	−179.9 (2)
C15—N1—C1—C2	49.3 (2)	C23—C22—C21—C20	0.5 (3)
S1—N1—C1—C2	−96.70 (17)	C9—C8—C13—C12	−0.3 (3)
C15—N1—C1—C6	−130.40 (16)	S1—C8—C13—C12	178.28 (18)
S1—N1—C1—C6	83.60 (17)	C5—C6—C7—O1	11.4 (3)
C26—C23—C18—C19	−178.47 (18)	C1—C6—C7—O1	−170.5 (2)
C22—C23—C18—C19	1.4 (3)	C12—C11—C10—C9	0.0 (3)
C26—C23—C18—C17	−2.9 (3)	C14—C11—C10—C9	179.0 (2)
C22—C23—C18—C17	177.00 (16)	C8—C9—C10—C11	0.2 (3)
C6—C1—C2—C3	−0.9 (3)	C23—C18—C19—C20	0.7 (3)
N1—C1—C2—C3	179.41 (17)	C17—C18—C19—C20	−174.9 (2)
C24—C16—C17—C18	171.62 (16)	C1—C2—C3—C4	−1.1 (3)
C15—C16—C17—C18	−7.0 (3)	C21—C22—C29—C28	−177.0 (2)
C19—C18—C17—C16	−58.0 (3)	C23—C22—C29—C28	2.6 (3)
C23—C18—C17—C16	126.4 (2)	C1—C6—C5—C4	−2.3 (3)
C13—C8—C9—C10	0.0 (3)	C7—C6—C5—C4	175.8 (2)
S1—C8—C9—C10	−178.60 (15)	C23—C26—C27—C28	1.5 (3)
C1—N1—C15—C16	67.76 (17)	C22—C21—C20—C19	1.5 (4)
S1—N1—C15—C16	−146.88 (12)	C18—C19—C20—C21	−2.2 (4)
C17—C16—C15—N1	−108.93 (19)	C22—C29—C28—C27	−1.4 (4)
C24—C16—C15—N1	72.38 (17)	C26—C27—C28—C29	−0.6 (4)
C2—C1—C6—C5	2.6 (3)	C10—C11—C12—C13	−0.3 (4)
N1—C1—C6—C5	−177.71 (17)	C14—C11—C12—C13	−179.4 (2)
C2—C1—C6—C7	−175.45 (18)	C8—C13—C12—C11	0.5 (4)
N1—C1—C6—C7	4.3 (3)	C6—C5—C4—C3	0.3 (4)
C25—O5—C24—O4	3.5 (3)	C2—C3—C4—C5	1.4 (4)

### Hydrogen-bond geometry (Å, °)

**Table:**

Cg is the centroid of the C22/C23/C26–C29 ring.

**Table:**

*D*—H···*A*	*D*—H	H···*A*	*D*···*A*	*D*—H···*A*
C25—H25A···O4^i^	0.96	2.50	3.462 (3)	177.
C10—H10···O2^ii^	0.93	2.44	3.305 (2)	155.
C17—H17···Cg^iii^	0.93	2.78	3.528 (2)	138.

Symmetry codes: (i) −*x*+1, −*y*+1, −*z*+2; (ii) *x*−1, *y*, *z*; (iii) −*x*, −*y*, −*z*+2.
